# Detectable HIV-RNA in semen of HIV controllers

**DOI:** 10.1371/journal.pone.0183376

**Published:** 2017-08-16

**Authors:** Marie-Laure Chaix, Faroudy Boufassa, Candice Meyzer, Marianne Leruez-Ville, Nadia Mahjoub, Marie-Laure Nere, Philippe Genet, Claudine Duvivier, Caroline Lascoux-Combes, Olivier Lambotte, Jade Ghosn

**Affiliations:** 1 APHP, Laboratoire de virologie, Hôpital Saint Louis, Paris, France; 2 INSERM U941, Université Paris Diderot, Paris, France; 3 INSERM CESP U1018, Faculté de Médecine Paris Sud, Le Kremlin-Bicêtre, France; 4 Centre d’Investigation Clinique Mère-Enfant, Hôpital Necker-Enfants Malades, Paris, France; 5 APHP, Laboratoire de Virologie, Hôpital Necker-Enfants Malades, Paris, France; 6 Centre hospitalier Victor Dupouy, Service d’hématologie, Argenteuil, France; 7 Institut Pasteur, et Centre Médical Necker-Pasteur, Paris, France; 8 APHP, Hôpital Saint Louis, service de maladies infectieuses, Paris, France; 9 APHP, Service de Médecine Interne et d’Immunologie Clinique, Hôpital Bicêtre, Le Kremlin Bicêtre, France; 10 INSERM, U1184, Immunology of Viral Infections and Autoimmune Diseases, Le Kremlin-Bicêtre, France; 11 Université Paris Sud, UMR 1184, Le Kremlin-Bicêtre, France; 12 CEA, DSV/iMETI, IDMIT, Fontenay-aux-Roses, France; 13 Université Paris Descartes, Paris, France; 14 INSERM UMR-S 1136, Institut Pierre Louis d’Epidémiologie et de Santé Publique, Université Pierre et Marie Curie, Paris, France; Hôpital Bichat-Claude Bernard, FRANCE

## Abstract

**Background:**

Whether spontaneous low levels of HIV-1 RNA in blood plasma correlate with low levels of HIV-1 RNA in seminal plasma has never been investigated in HIV controller (HIC) men so far.

**Methods:**

HIC men enrolled in the ANRS CODEX cohort were eligible for the present study if they had no symptoms of sexually transmitted infections (STI). Two paired samples of blood and semen were collected four weeks apart. HIV-RNA was quantified in blood plasma (bpVL) and in seminal plasma (spVL), and cell-associated HIV-DNA was quantified in peripheral blood mononuclear cells (PBMC) and in non-sperm cells (NSC). Spearman rho tests were used to estimate correlations between bpVL and spVL.

**Results:**

Ten men were enrolled. At Day 0 (D0), spVL was detectable in four patients: 458; 552; 256 copies/mL and PCR signal detectable below limit of quantification (LoQ, 40 copies/mL). At Day 28 (D28), spVL was detectable in the same four participants in whom spVL was detectable at D0 with 582; 802; 752 and 50 copies/mL, respectively. HIV-DNA was detectable below LoQ in NSC of one patient at D0 visit. No patient had detectable HIV-DNA in NSC at D28 visit. At D0, bpVL and spVL were highly positively correlated (Spearman rho: 0.94; p = 0.0001). Similar results were found at D28.

**Conclusion:**

We show that HIV-RNA can be detected in the semen of HIC men, with levels positively correlated with those measured concomitantly in blood plasma. HIC men should be aware of the risk of HIV genital shedding, especially if viral blips are reported.

## Introduction

Combined antiretroviral therapy (cART) is able to achieve HIV-RNA suppression in blood plasma (bpVL) and subsequently reduce the levels of seminal plasma HIV-RNA (spVL) in the vast majority of treated patients [1, 2]. This is a major finding supporting universal cART as “treatment as prevention” [3]. Interestingly, a significant proportion (5–30%) of men living with HIV on successful cART may still harbor detectable levels of HIV-RNA in seminal plasma [4, 5]. HIV controllers (HIC) are individuals in whom HIV-1 is sustainably low without any cART, regardless of the CD4 cell count [6]. Whether spontaneous low levels of bpVL in HIC correlate with low levels of spVL has never been investigated so far, while it has been addressed in HIC women [7], showing absence or very low levels of HIV-RNA in cervico-vaginal lavage samples. Here we quantified HIV-RNA in seminal plasma and cell-associated HIV-DNA in non-sperm cells (NSC) of HIC men enrolled in the ANRS CODEX cohort [8].

## Patients and methods

### Study design and study participants

HIV-1 controllers (HIC) were identified from the French CO21-CODEX cohort which has included HIV-1 controllers fulfilling the ANRS definition, i.e. patients with a minimum of 5 bpVL measurements below 400 copies/mL over a minimum of 5 years despite never receiving ART [6, 9]. All available measures of bpVL, CD4 and CD8 T cell counts and their percentages since the date of HIV-1 diagnosis are collected. Period of control was defined as the first time, among all available measures, bpVL dropped below 400 copies/mL. During the period of control, a viral blip was defined as a measure of isolated bpVL above 400 copies/mL returning to <400/mL. A unique measure of VL above 2000 copies/mL or a drop of CD4 cell count below 350/mm^3^ was considered as a suspected escape of control. Two consecutive bpVL measures above 2000 copies/mL or two consecutive CD4 T cell measures below 350/mm^3^ were considered as a confirmed escape of control. The present study included 10 HIC men enrolled in the cohort. All patients were seen at baseline (D0) and at day 28 (D28) at the Centre d’Investigations Cliniques (CIC) of Necker Hospital for clinical examination and for collection of paired samples of blood and semen. Sexually transmitted infections (STI) were systematically looked for and patients were excluded if having symptoms of STI. The Ethics Committee “Ile de France VII” approved the present study and all participating patients signed a specific written informed consent prior to enrolment.

### Assessment of blood and semen samples

Semen samples were collected in sterile containers by self-masturbation after a recommended 48-hour abstinence period then sent, within 4 hours of collection, to the Virology Laboratory. Quantification of HIV-1 RNA in blood plasma and seminal plasma was performed using COBAS® AmpliPrep/COBAS® TaqMan® HIV-1 Test v2.0 (Roche Diagnostics, Meylan, France) with a threshold of 20 copies/mL for blood. To avoid PCR inhibition in semen, a dilution (1/2 or 1/3) was performed. The threshold was 40 or 60 copies/mL according to the dilution. NSC were harvested as described elsewhere [10]. Cell-associated HIV-DNA was quantified in peripheral blood mononuclear cells (PBMC) and in NSC at D0 and D28 as described elsewhere [11, 12].

### Statistical analysis

Demographic and immuno-virological characteristics at the time of measurements were described by the median and interquartile range for continuous variables, and frequency and percentages for discrete variables. For the main analysis and when HIV RNA were below the LoQ, the HIV RNA values were set to half of the threshold if the measure was strictly undetectable (i.e. if the result of the measure was undetectable but <20 copies/mL the HIV RNA was set to 10 copies/mL) and when the measure was not strictly undetectable the values were set to the threshold (i.e. if the result of the measure was detectable but <20 copies/mL the HIV RNA was set to 20 copies/mL) according to the decision made by the virological laboratory. In the sensitive analyses, we chose two strategies. First, when HIV RNA were below the LoQ, we set the values of samples under the threshold to 1 if the measure was strictly undetectable (i.e. if the result of the measure was undetectable but <20 copies/mL the HIV RNA was set to 1 copies/mL) and to a range of randomized values between 1 and the threshold minus 1 otherwise (i.e. if the result of the measure was detectable but <20 copies/mL we set a randomised values between 1 (which is the minimum possible value) and 19 (which is the minimum possible value if the threshold was 20 copies/mL)). Secondly, we categorised the values of HIV RNA with the values of 0 if the values of HIV RNA were undetectable (strictly or not strictly) and 1 if the values were detectable. The main and the sensitive analyses yielded same results. Spearman rho tests were used to estimate correlations between bpVL and spVL or HIV-1 DNA in PBMC and HIV-1 DNA in NSC in each period separately and within periods of measurements. Fisher’s exact tests were performed to compare detection of spVL and bpVL in each period and to compare qualitative characteristics of patients at D0. Wilcoxon rank sum tests were used to compare quantitative characteristics at D0. STATA software (Version 14.0, Stata Corp., College Station, Texas) was used for statistical analysis.

## Results

### Patients’ characteristics at baseline visit (D0)

Ten men were enrolled between December 2013 and October 2014; their main characteristics being: median time since HIV diagnosis was 11 years (8–18), median time of control 10.3 years (8–18), median age 45 years (IQR: 39–55) median CD4 698/mm^3^ (640–745), median CD8 785/mm^3^ (704–1023) and median CD4/CD8 ratio 0.89 (0.61–1.21). None had symptoms suggestive of STI. During the period of control, 4 patients had no blip, 4 patients had only 1 blip, 1 patient had 3 blips and the last one had 4 blips. The two highest blips were 1965 copies/mL in one patient and 1194 copies/mL in the other one. These two patients returned to a bpVL below 400 copies/mL thereafter. No patient had a suspicion or a confirmation of escape.

### Detection of HIV-RNA in bpVL and in spVL

Results are summarized in Table 1. At D0, nine out of the ten patients had a detectable bpVL: five had a bpVL < 400 copies/mL and the remaining four had a bpVL of 685; 5757; 1003 and 468 copies/mL. spVL was detectable in four patients: 458; 552 and 256 copies/mL in the first three participants, the remaining participant having a spVL PCR signal detectable but below LoQ (40 copies/mL).

**Table 1:** Quantification of HIV-RNA and HIV-DNA in blood and in semen of HIV-controller men.

Patient	First samples					Second samples				
	Time of sample	Bp HIV RNA	Sp HIV RNA	HIV DNA in PBMC	HIV DNA in NSC	Time of sample	Bp HIV RNA	Sp HIV RNA	HIV DNA in PBMC	HIV DNA in NSC
		copies/ml	copies/ml	copies /10^6^ PBMC	copies /10^6^ cells		copies/ml	copies/ml	copies /10^6^ PBMC	copies /10^6^ cells
18001	12/10/2013	<20	<40	472	<50	01/10/2014	D<20*	<40	400	<50
18007	02/13/2014	91	<60	<25	<50	03/24/2014	42	<40	25	<50
34020	08/19/2015	51	<40	32	<50	09/29/2015	325	<60	25	<50
48001	01/21/2014	685	458	32	D<50**	02/27/2014	347	582	25	<50
48003	03/19/2014	D<20*	<40	50	<50	04/29/2014	78	<40	60	<50
56007	07/24/2014	5757	552	280	<50	08/28/2014	596	802	500	<50
57003	01/29/2015	1003	256	100	<50	03/05/2015	No sample	752	60	<50
63009	05/07/2014	37	<40	80	<50	06/12/2014	D<20*	<40	130	<50
63014	10/06/2015	D<20*	<40	12	<50	11/18/2015	75	<40	12	<50
73010	05/09/2014	468	D<40	80	<50	06/26/2014	375	50	60	<50

Bp: blood plasma, sp: seminal plasma, PBMC: peripheral blood mononuclear cells, NSC: non sperm cells

*D<20: detectable but below the threshold of the technique (20 RNA copies/mL)

**D<50: detectable but below the threshold of the technique (50 DNA copies/10^6^ cells)

At D28, nine patients had a detectable bpVL (8/9 < 400 copies/ml) and bpVL could not be quantified in the remaining participant due to technical issues. At D28, spVL was detectable in the same four participants (and no additional ones) in whom spVL was detectable at D0: 582; 802; 752 and 50 copies/mL, respectively.

### Quantification of cell-associated HIV-DNA in PBMC and in NSC

HIV-DNA was detectable in PBMC in 10/10 patients at D0 and 9/10 at D28, with a median level of 65 copies/10^6^ PBMC (range 12–472) and 60 copies/10^6^ PBMC (range 12–500) at D0 and D28, respectively. HIV-DNA was detectable (but below LoQ) in NSC of one patient at D0 visit. This patient had a concomitant spVL of 458 copies/ml. No patient had detectable HIV-DNA in NSC at D28 visit (Table 1).

### Correlations

The four patients who had a detectable spVL at D0 also had a concomitant detectable bpVL, with a bpVL > 400 copies/ml in all four of them (Table 1). At D0, HIV-1 RNA in plasma and HIV-1 RNA in seminal plasma were highly positively correlated (Spearman rho: 0.94; p = 0.0001). No significant correlations were found between HIV-1 RNA in blood plasma and HIV-1 DNA in PBMC (rho 0.12; p = 0.74), HIV-1 RNA in seminal plasma and HIV-1 DNA in PBMC (rho 0.20; p = 0.58) and HIV-1 DNA in NSC and HIV-1 DNA in PBMC (rho 0.43; p = 0.21).

One out of the four patients who had a detectable spVL at D28 had a bpVL > 400 copies/mL (Table 1). At D28, we found similar results with a very high positive correlation between HIV-1 RNA in blood plasma and HIV-1 RNA in seminal plasma (rho 0.90; p = 0.001) and no correlation between other measures.

Between periods, we found a good correlation between the two measures of HIV-1 RNA in blood plasma (rho 0.77; p = 0.02) and the two measures of HIV-1 RNA in seminal plasma (rho 0.86; p = 0.001).

At D0, neither detectable HIV-1 RNA in blood plasma nor detectable HIV-1 RNA in seminal plasma were associated with viral blips during period of control (Fisher exact test p = 0.50 and 0.33, respectively). Duration of control (before D0) was also not associated with detectable HIV-1 RNA in blood plasma (p = 0.31).

## Discussion

To the best of our knowledge, this is the first study addressing the levels of HIV-RNA and cell-associated HIV-DNA in semen of HIC men. We show here that HIV-RNA can be detected in the semen of HIC men, with levels positively correlated with those measured concomitantly in blood plasma. As HIV-RNA shedding in semen has been previously found to be intermittent [5], we collected two paired samples of blood and semen four weeks apart, and results were very consistent at each period. Local viral production in the male genital tract may be triggered by the concomitant presence of symptomatic STI [13]. None of the participants in our study, however, had symptoms suggestive of STI.

Interestingly, we show that HIC men can have transient high levels of bpVL without displaying a confirmed escape of control in blood plasma. Patient 56007 had a bpVL reaching 5757 copies/mL at D0, and a spontaneous one log_10_ drop at D28 (596 copies/mL). Such blips can occur in HIC patients [6, 8]. This finding emphasizes the importance of measuring HIV-RNA always with the same commercial kit for consistency of the results [14]. Indeed, the last bpVL measured for patient 56007 in his clinical center, just before he was enrolled in the study, was 100 copies/mL using a different commercial kit from the one used in the present study (data not shown). In our study, the history of blips did not predict the detection of HIV-RNA in semen, which is most probably due to a lack of power.

HIV-DNA levels in PBMC were low and stable between the two visits. This is in keeping with previous results in HIC [8, 15]. HIV DNA in NSC was detected in only one patient with concomitant detectable HIV-RNA in blood plasma and in seminal plasma. Gantner et al. showed that HIV-DNA was detected in 25 out of 160 NSC samples (16%; median: 21 copies/10^6^ NSC) in men who have sex with men on successful cART with sustained suppression of HIV-RNA in blood plasma [12]. In the latter study, median HIV-DNA was PBMC was 229 copies/10^6^ PBMC [5]. Inconsistent and low detection of HIV-DNA in NSC of HIC men could be explained by low levels of HIV replication in blood plasma and the very low levels of HIV-DNA in PBMC, together with intrapatient dynamics of HIV in the genital reservoir.

HIV-RNA levels in seminal plasma ranged between 50 and 802 copies/mL. The question that remains unanswered is whether such levels of HIV-1 RNA are infectious, ie, whether there is a transmission threshold. Of note, the four patients with detectable spVL at D0 also had detectable spVL at D28. Such continuous spVL shedding suggests different mechanisms of local viral persistence in HIC men than in cART-treated men [5, 12]. Indeed, unlike the findings in HIV-infected MSM with sustained full viral suppression in blood plasma [5, 12], the continuous genital shedding in HIC men, together with the strong correlation between spVL and bpVL and the absence of detection of HIV-DNA in NSC, suggests that spVL originates from passive diffusion from blood plasma rather than compartmentalization with local viral production in the male genital tract.

The small sample size is a limitation of our study, but not only the HIC population is very small among people living with HIV; also, the proportion of women is higher among the HIC population [15]. Overall, at present, there is insufficient evidence to make a general claim that HIC men are not infectious. HIC men should be aware of the risk of HIV genital shedding, especially if viral blips are reported. Further studies are needed to better understand the origin and cause of viral shedding in semen of HIC men.
